# Otitis media with effusion in children aged 2–12 years attending the paediatric clinic at Mulago National Referral Hospital, a Ugandan tertiary hospital: a cross-sectional study

**DOI:** 10.1186/s12887-022-03408-w

**Published:** 2022-06-22

**Authors:** Kabagambe Bamaraki, Justine Namwagala, Rym Hidour, Emma Nsalazi Bambi

**Affiliations:** 1 Department of Ear, Nose, and Throat, Makerere University, College of Health Sciences, Kampala, Uganda; 2 Department of Pediatrics and Child Health, Makerere University, College of Health Sciences Kampala, Kampala, Uganda

**Keywords:** Otitis media with effusion, Hearing loss, Children, Prevalence, Associated factors

## Abstract

**Background:**

Otitis media with effusion (OME) is common in children aged between 6 months to 4 years, and it is one of the causes of hearing loss (HL) in children worldwide. OME is a type of inflammation of the middle ear in which there is a collection of fluid. The latter causes HL which interferes with speech and language development, communication skills, school performance, psychosocial skills, and quality of life of children.

**Methods:**

This was a prospective cross-sectional study on 246 children aged 2 -12 years, attending the Mulago National Referral Hospital (MNRH). A consecutive sampling procedure was used to reach each participant under ethical considerations until the sample size was reached. All children aged 2–12 years who meet inclusion criteria were examined first by the Pediatrician and then by the Principal Investigator. Patients with tympanogram type B (flat curve) were diagnosed to have OME. The prevalence of OME was summarized as a proportion and multivariate analysis was used to determine the factors associated with OME. Data were analyzed using the STATA version 13.0.

**Results:**

A total of 246 children were recruited for the study. Of the 246 children, 60% were male. The median age of the participants was 4.8 ± 2.8 years. The prevalence of OME was found to be 11%. Upper respiratory tract infections (URTI), recurrent AOM (*p* = 0.005, OR:5.14, 95% CI: 1.66–15.96), and snoring (*p* = 0.000, OR: 6.32, 95% CI: 2.32–17.26) were found to be strongly associated with OME in children aged 2–12 years attending the Mulago National Referral Hospital.

**Conclusions:**

The prevalence of OME among children aged 2–12 years attending MNRH was found to be 11%. There is an association between OME and URTI, recurrent AOM, and snoring in children aged 2–12 years attending MNRH.

## Background

Otitis media with effusion (OME) is a type of inflammation of the middle ear in which there is a collection of fluid without symptoms or signs of acute ear infection. It is mostly due to a blockage or dysfunction of the Eustachian tube (ET), most often between the ages of 6 months and 4 years. This later causes HL which interferes with speech and language development, communication skills, school performance, psychosocial skills, and quality of life of children [1–3].

Otitis media with effusion is one of the causes of hearing loss (HL) in children worldwide. Otitis media with effusion is one of the most commonly occurring childhood illnesses in the United States of America (USA), with more than 2.2 million diagnosed cases each year [4].

The prevalence of OME is reported in the developed countries, USA 15 to 30% in adolescents [5], 63.9% in Italy in children with lower respiratory tract infection [5].

In developing countries, India with 32.3% [6], and in African countries, South Africa has a prevalence of 16.5% in children attending a primary health care clinic [7], Nigeria with 25.2% of school-age children [8], and Uganda 14.1% of school-going children [9].

Otitis media with effusion (OME) remains a public health problem around the world. In Uganda, there are unpublished data about the prevalence and risk factors of otitis media with effusion in the Paediatric clinic at MNRH. It was, therefore, important to conduct this study for this age group (2–12- year-old) to determine the prevalence and the associated factors of otitis media with effusion in children aged 2–12 years attending the Paediatric Clinic at MNRH.

## Methods

### Study design, setting, and population

This was a prospective cross-sectional study carried out among children aged 2 to 12 years, attending the Paediatric Clinic at the Mulago National Referral Hospital, Kampala, Uganda from February 2019 to April 2019. The inclusion criteria were all children aged 2–12 years, attending the Paediatric clinic at MNRH, all children whose parents or guardians consented, and all children with ages more than 8 years who assented to the study. The exclusion criteria were children with chronic ear infections, perforation of the tympanic membrane; children who were sick and needed to be admitted, and uncooperative children.

### Study procedure

Recruitment of participants was done at the Paediatric Clinic, Mulago National Referral Hospital. Consecutive sampling was used on those who satisfy the eligibility criteria until the sample size was achieved. The research assistants and/or the principal investigator comprehensively explained to the parents or caretakers of the selected participants the purpose of the study, benefits, and risks and thereafter requested their participation. The eligible patients who agreed to participate signed a consent/assent form by writing their names and signature and subsequently were assigned a student number. Participants were then interviewed after seeing the Paediatrician or Clinician. They were examined and underwent otoscopy and tympanometry by the PI assisted by an Audiologist and the Pediatrician in the consultation room of the Pediatric Clinic. The diagnosis of OME was confirmed following the otoscopy findings (dull, retracted eardrum, presence of an air bubble or air-fluid level) and tympanometry findings (tympanogram type B).

We do not currently have at the Paediatric Clinic facilities to provide behavioral hearing testing in children, such as play audiometry and visual reinforcement audiometry, ABR, so our hearing assessment is limited to otoscopy and tympanometry.

### Otological exam

Otoscopy was performed by the PI using a Welch-Allyn otoscope. This included the status of the tympanic membrane, normal, dull, retracted, presence of an air bubble or air-fluid level, absence or presence of perforation with or without pus discharge. The children who didn’t have wax, tympanic membrane perforation, or ear discharge, were sent to the next test which was the tympanometry. Participants with impacted wax were advised to use a cerumen softener for three days. After the use of the softener, the ear syringing was carried out, then followed by an audiological assessment.

### Statistical data analysis

Analysis of the data collected was done using a computer statistical software EPI-DATA version 3.1 with automatic checks to avoid double entries of a questionnaire. The entered data were then exported to STATA version 13.0 for data cleaning and analysis with the help of a professional statistician. Continuous variables were analyzed using the mean, standard deviations, median and interquartile ranges while categorical variables were presented using tables, bar graphs, and pie charts.

The prevalence of OME in children aged 2–12 years was determined using simple proportions, i.e., by calculating the number of children with OME 2–12 years divided by the total number of children recruited into the study. The associated factors were assessed in steps: bivariate analysis followed by multivariate analysis, with variables with a *p*-value < 0.2 at bivariate analysis being included in the multivariate model, using logistic regression. The results were presented using odds ratios and *P*-values whereby, the *P*-value of 0.05 was used as the measure of statistical significance when considering 95% confidence intervals.

### Ethical approval

A permission letter to carry out the study was obtained from the ENT Department, College of Health Sciences (CHS), Makerere University, School of Medicine Research and Ethics Committee (SOMREC). A written informed consent/assent was sought from parents/caretakers/children before recruitment into the study, and it included a clear explanation of the purpose of the study, and the possible benefits and risks.

## Results

Between February 2019 to April 2019, a total of 256 children attending the Paediatric Clinic at Mulago Hospital were invited to participate in the study; 4 caretakers decline to consent, 2 children were outside the required age range of 2 to 12 years, 4 were unable to participate due to other reasons and 246 were recruited**.**

### Demographic, profile, and clinical characteristics of the study participants

Table 1 below shows the socio-demographic characteristics of the study participants (Table 1).

**Table 1:** Shows socio-demographic characteristics of study participants

Characteristic		Frequency (*n* = 246)	Percentage (%)
**Age in years (Mean ± SD*)***	2–4	154	63
	5–7	53	21
	8–10	22	9
	11–12	17	7
**Sex of the child**	Male	147	60
	Female	99	40
**School attendance**	Yes	173	70
	No	73	30
**Breastfeeding duration**	Never	1	1
	< 6 months	5	2
	6–12 months	26	10
	> 12 months	214	87
**Breastfeeding position**	Head raised	238	97
	Flat	8	3
**Mothers’ education**	No/low education	90	37
	Higher/Tertiary	156	63
**Family size**	≤ 3 people	38	15
	4–6	164	67
	≥ 7	44	18

**Fig. 1:**
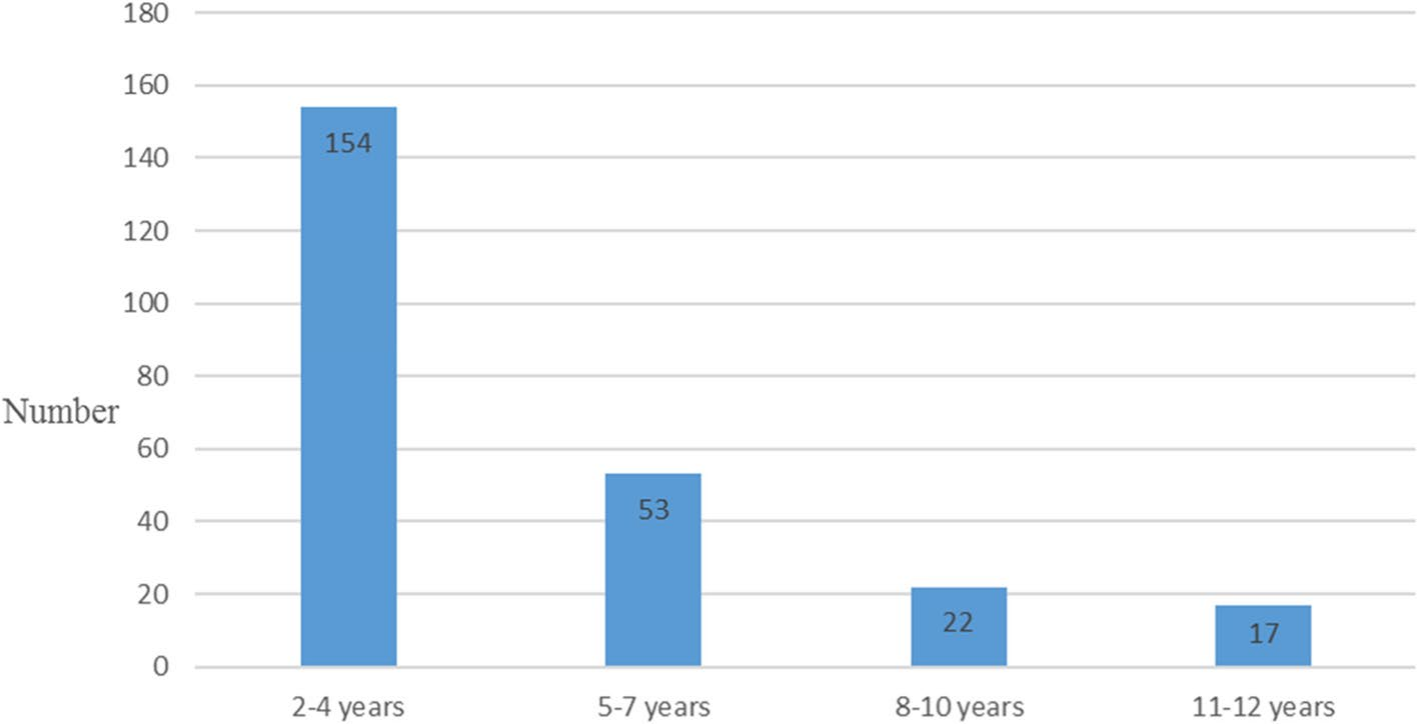
Shows the age distribution of the study participants

Among the 246 children recruited aged 2–12 years, the mean age of the participants was 4.8 ± 2.8. Most of the participants 154/246 (63%) were aged 2–4 years while 22/246 (9%) were within the age group of 8–10 years and 17/246 (7%) were within the age group of 11–12 years (Fig. 1). Overall, 147/246 (60%) of the children were male. The histogram below shows the age distribution of the study participants (Fig. 1).

### Prevalence of OME in children aged 2–12 years attending the Paediatric Clinic at MNRH

**Fig. 2:**
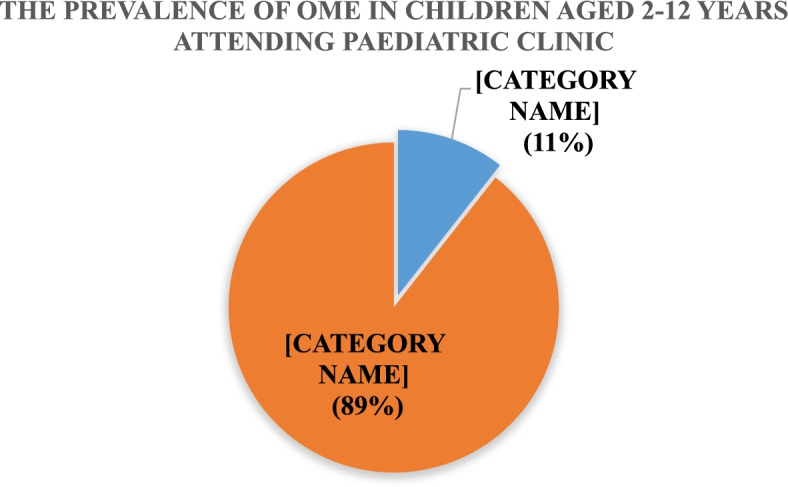
Pie chart showing the prevalence of OME in children aged 2–12 years attending the Paediatric Clinic

Among the 246 children recruited aged 2–12 years, the prevalence of OME in this study was 11% (26/246) as shown in Fig. 2 below (Fig. 2). Children without OME were 220/246 (89%). All 26 patients with OME presented bilateral OME.

### Factors associated with OME among children aged 2–12 years attending the Paediatric Clinic

Using the bivariate analysis, the table below shows history of frequent AOM (*p* < 0.001, OR:6.75, 95% CI: 2.60–17.54); allergy (*p* = 0.007, OR:3.24, 95% CI:1.38–7.61), snoring (*p* < 0.001, OR:7.66, 95% CI:3.23–17.18), delayed speech (*p* = 0.005, OR:3.67, 95% CI:1.48–8.93) were significantly associated with OME. Important to know also is that all 26 children who had a tympanogram type B for both ears, had a history of frequent URTI. Other factors like age, sex, school attendance, mothers’ education, family size, and exposure to smoking were not directly associated with OME as shown in the table below (Table 2):

**Table 2:** Shows bivariate analysis of socio-demographic characteristics with OME in children aged 2–12 years attending the Paediatric Clinic at MNRH

Characteristics	*(Mean* ± *SD)*	Had Type “B” (*n* = 26) *3. 3* ± *1. 6*	No type B (*n* = 220) 4. 9 ± 2. 9	OR (95% CI)	*P*-value
Age in years	2–4	24 (92)	130 (59)	0.26 (0.03 – 2.01)	0.196
	5–7	1 (4)	52 (24)	2.48 (0.15 – 41.45)	0.528
	8–10	1 (4)	21 (10)	-	-
	11–12	0 (0)	17 (9)	Ref	
Sex	Male	17 (65)	130 (59)	1.31 (0.56 – 3.06)	0.537
	Female	9 (35)	90 (41)	Ref	
School attendance	Yes	21 (81)	152 (69)	1.88 (0.68 – 5.19)	0.224
	No	5 (19)	68 (31)	Ref	
Mothers’ education	No/low education	11 (42)	79 (36)	1.31 (0.57 – 2.99)	0.523
	Higher/Tertiary	15 (58)	141 (64)	Ref	
Family size	≤ 3 people	2 (8)	36 (16)	Ref	
	4–6	20 (77)	144 (66)	0.40 (0.09 – 1.79)	0.231
	≥ 7	4 (15)	40 (18)	0.56 (0.10 – 3.22)	0.512
Exposure to smoking	Yes	1 (4)	9 (4)	0.94 (0.11 – 7.71)	0.952
	No	25 (96)	211 (96)	Ref	
**Frequent AOM**	Yes	9 (35)	16 (7)	6.75 (2.60 – 17.54)	** < 0.001**
	No	17 (65)	204 (93)	Ref	
**Allergy**	Yes	17 (65)	81 (37)	3.24 (1.38 – 7.61)	**0.007**
	No	9 (35)	139 (63)	Ref	
**Snoring**	Yes	16 (62)	38 (17)	7.66 (3.23 – 17.18)	** < 0.001**
	No	10 (38)	182 (83)	Ref	
**Delay speech**	Yes	9 (35)	28 (13)	3.67 (1.48 – 8.93)	**0.005**
	No	17 (65)	192 (87)	Ref	

Considering the multivariate analysis, the prevalence in affected children was strongly associated with recurrent AOM (*p* = 0.005, OR: 5.14, 95% CI: 1.66–15.96), snoring (*p* = 0.000, OR: 6.32, 95% CI:2.32–17.26). Important to note all 26 children with OME had URTI and reported snoring at night. This is possibly due to enlarged adenoids, which could affect the function of the Eustachian tube.

Sex, school attendance, exposure to smoking, allergy, and delayed speech were less associated with OME as shown in Table 3 below (Table 3):

**Table 3:** Shows a multivariate analysis of the Factors associated with OME in children aged 2–12 years attending the Paediatric Clinic at MNRH

**Characteristics**	**odds ratio**	**95% CI**	Type="Bold">*P*-value*
Male child	2.22	0.80 – 6.20	0.127
School attendance	2.78	0.82 – 9.44	0.102
Exposure to smoking	0.22	0.01—3.23	0.269
**Frequent AOM**	**5.14**	**1.66 – 15.96**	0.005*
Allergy	1.49	0.56 – 3.99	0.429
**Snoring**	**6.32**	**2.32 – 17.26**	0.000*
Delayed speech	1.99	0.64 – 6.20	0.235

^*^Statistically significant risk factors

## Discussion

This study set out to determine the prevalence and associated factors of otitis media with effusion among children aged 2–12 years attending the Paediatric Clinic at MNRH.

### Prevalence of otitis media with effusion

The prevalence of otitis media with effusion in this study was found to be 11%. All 26 children with OME had bilateral OME, there were no children with unilateral OME. This is similar to the prevalence of OME (11.9%) found in a study done by Els, et al. in South Africa, in 2016 [10], where 109 children aged 2–12 years were recruited.

Also, a study done by Caylan R, et al., in Turkey, in 2006, found the prevalence of OME was 11.1% in children aged 5–12 years. This study had similar findings to our study, even though they didn’t have in their studies, children younger than 5 years [11].

In another cross-sectional study done by Chibuike, et al., in Nigeria, in 2017 [8], the prevalence of OME was reported to be 25.2% in children aged 1 to 6 years. In this study, the prevalence of OME was higher than what we found in our study. This could be attributed to the fact that it was a community study conducted in the Subregion of Nigeria, which is characterized by high rainfall and humidity. It is also known as an industrial area with high air pollution. Another reason could be they considered tympanogram types B and C as diagnoses of OME.

Aydemir G, et al., in Turkey, 2011 [12], found that 16% of 423 children 7–12 years enrolled had OME. This prevalence was higher as well compared to ours, the reason may be because they considered the types B and C tympanograms as indicating OME. In our study, the diagnosis of OME was based on a type B tympanogram (flat curve).

Bisso F (unpublished data), in Uganda, in 2002 [9], found the prevalence of OME was 14.1% of school-going children aged 4–6 years. This prevalence was slightly similar to our study, the only difference between, the former was a community study and didn’t include children less than 4 years and older than 6 years.

### Factors associated with otitis media with effusion

We found frequent episodes of URTI, recurrent acute otitis media, and snoring to be factors strongly associated with otitis media with effusion, this is similar to that reported by F. Martines, et al. [13], where snoring (*p* < 0.0001; OR = 3.86, 95% CI: 2.72–5.47), recurrent acute otitis media (*p* < 0.0015; OR = 2.54, 95% CI: 1.41–4.61), frequent URTI (*p* < 0.0001; OR = 3.17, 95% CI: 2.22–4.52) were factors associated with otitis media with effusion.

Our results were also similar to the findings of Kiris M., et al. [14] who found that snoring (*p* < 0.0001) and URTI (*p* < 0.0001 were important associated factors with otitis media with effusion. But they didn’t consider recurrent acute otitis media as a variable, which was included in our present study.

In a study done by Aydemir G., et Al., in 2011 [12], URTI (*p* < 0.05), snoring (*p* < 0.05), and frequent episodes of acute otitis media (*p* < 0.05) were reported to be predictors of otitis media with effusion, which are similar to the findings of our study.

Snoring reported in our 26 children with bilateral OME could be due to a mechanical obstruction of the nasopharynx by an enlarged adenoid, which could affect the function of the Eustachian tube.

### Limitations of the study

Otitis media with effusion leads to conductive hearing loss in affected children, we would have wished to assess the degree, types, and configuration of the hearing loss, using the diagnostic Auditory Brainstem Response machine, but we were not able to do it.

Given how common OME is in our pediatric population, it would be very beneficial if the Audiologists of Mulago National Referral Hospital could be provided with the equipment and training necessary to perform behavioral hearing testing in children attending the Paediatric Clinic at MNRH. It is very important to diagnose and treat hearing loss in children to optimize their communication and education.

## Conclusion

The prevalence of OME in children aged 2–12 years attending the Paediatric Clinic at MNRH was found to be 11%, which is low or high compared to other studies.

Upper respiratory tract infections, recurrent acute otitis media, and snoring are found to be predictors of OME in the Pediatric Clinic at MNRH. We recommend to all Pediatricians and other medical professionals working at the Paediatric unit to always give special attention to children presenting with URTI and other ENT conditions such as hearing loss, recurrent acute otitis media, and snoring at night. Those who are not responding to the treatment should be referred early to the ENT Clinic for further management. The government should make policies on tympanometric tests at the Paediatric Clinic at MNRH, Kampala, Uganda for early detection of hearing loss in children, which could indicate the presence of OME, thus preventing them from sequelae that may arise.

## Data Availability

The datasets used and/or analyzed during the current study are not publicly available due to legal and ethical reasons but are available from the corresponding author on reasonable request.

## Abbreviations

ABR: Auditory Brainstem Response
AOM: Acute Otitis Media
CHL: Conductive Hearing Loss
CHS: College of Health Sciences
dB: Decibel
dBHL: Decibel Hearing Level
ENT: Ear, Nose and Throat
EAC: External Auditory Canal
HL: Hearing Loss
MoH: Ministry of Health
MNRH: Mulago National Referral Hospital
OME: Otitis Media with Effusion
PTA: Pure Tone Audiometry
SNHL: Sensorineural Hearing Loss
SOMREC: School Of Medicine Research and Ethics Committee
TM: Tympanic Membrane
URTI: Upper Respiratory Tract Infection
